# Insights into breast cancer phenotying through molecular omics approaches and therapy response

**DOI:** 10.20517/cdr.2018.009

**Published:** 2019-09-19

**Authors:** Jose E. Belizario, Angela F. Loggulo

**Affiliations:** ^1^Department of Pharmacology, Institute of Biomedical Sciences, University of São Paulo, Avenida Lineu Prestes, 1524, São Paulo, CEP 05508-900, Brazil.; ^2^Department of Pathology, Paulista School of Medicine, Federal University of São Paulo, Rua Sena Madureira, 1500, São Paulo, CEP 04021-001, Brazil.

**Keywords:** Breast cancer, ERBB/HER, estrogen receptor, progesterone receptor, genomics, proteomics, epigenomics, endocrine and targeted therapy

## Abstract

Breast cancer is the most common cancer in the world. Despite advances in early detection and understanding of the molecular bases of breast cancer biology, approximately 30% of all patients with early-stage breast cancer have metastatic disease. Breast cancers are comprised of molecularly distinct subtypes that respond differently to pathway-targeted therapies and neoadjuvant systemic therapy. However, no tumor response is observed in some cases and development of resistance is most commonly seen in patients with heterogeneous breast cancer subtype. To offer better treatment with increased efficacy and low toxicity of selecting therapies, new technologies that incorporate clinical and molecular characteristics of intratumoral heterogeneity have been investigated. This short review provides some examples of integrative omics approaches (genome, epigenome, transcriptome, immune profiling) and mathematical/computational analyses that provide mechanistic and clinically relevant insights into underlying differences in breast cancer subtypes and patients’responses to specific therapies.

## Introduction

Cancer is defined as genetic disease and is molecularly characterized by accumulation of mutations and epimutations that lead to functional dysregulation of cell genome and epigenome-driven processes^[1]^. Nonetheless, different genetic and epigenetic variations within patients to patients can lead to a same disease phenotype, e.g., uncontrolled cell growth. Recent advances in whole genome throughput DNA sequencing, systems biology and machine learning algorithms have produced promise information on the evolutionary dynamics of tumors, from a single cell to a cell population^[2-4]^. The complex landscape of somatic modifications (copy number variations, mutations, gene rearrangements) observed in breast tumors are typically the result of a relatively small number of functional driver oncogenic alterations and a large number of non-functional passage alterations. The functional consequence of most cancer mutations could be characterized in phenotypic readouts such as growth assays or drug response screens using tumor cell lines^[5-7]^. The impact of mutations on kinase binding specificity has been explored *in vitro* protein binding microarrays, or *in vivo*, for example, by measuring kinase target phosphorylation after kinase mutation^[8]^. However, what substantially contribute to oncogenesis and progression of tumors is intra and inter-clonal heterogeneity, which is determined by a stochastic mutational process in cancer cells^[9,10]^.

Most common factors taken into account for classification of tumors into distinct groups include: degree of local invasion, degree of remote invasion, histological types of cancer and specific grading based on various tumor markers, and general status of the patient^[2,3]^. However, cancers with similar morphological and histopathological features reveal very distinct patterns of progression and response to therapy. With growing number of database of tumor and single cell genomic, epigenomic, and pharmacogenomics reporting on differences and similarities between clinical outcomes of patient cohort to the same therapies, we have a rewarding opportunity to modeling and predicting outcomes to traditional chemotherapy and targeted therapies^[11]^.

Epigenetic alterations participate in all steps of cancer, from tumor initiation to cancer progression and metastasis^[1,12]^. It is now well understood that both losses and gains of DNA methylation occurring in CpGs motifs, as well as aberrant chromatin organization, contribute significantly to cancer associated phenotypes^[12]^. Epigenetic inactivation of tumor suppressor genes (*TSGs*) is a well-established event in cancer progression. Oncogenic activating mutations are now known to occur in a number of epigenetic modifiers such as isocitrate dehydrogenase 1 and 2, histone-lysine N-methyltransferase enzyme (EZH2), DNA methyl transferases (DNMTs)^[13]^. In cancer cells, global DNA hypomethylation is frequently concomitant with both local hypo and hypermethylations (epigenetic mosaicism). This may contribute to cancer progression through chromosomal instability, reactivation of transposable elements and loss of imprinting. The DNMTs: DNMT1, DNMT2, and the DNMT3A/3B/3L are main components methylation machineries whereas demethylases Ten-eleven translocation proteins TET1, TET2 and TET3, and repressive histone-modifying enzymes of the Polycomb repressive complex 1 (PRC1) and PRC2, specifically named, “writers” and “erasers” are modulators of epigenetic patterns^[12]^. Defects in DNA methylation could induce TSG silencing and resistance to classical chemotherapeutic agents. Interplay between DNA methylation and DNA repair machineries may also occasionally provoke de novo methylation and aberrant gene silencing^[12-14]^. Methylome profiles provides a molecular signature of cancers and might serve as potential diagnostic and predictive biomarkers^[15-17]^. Some new epigenetic modifier drugs combined with conventional chemotherapies and immunotherapies may reverse more permanent changes that affect the epigenetic processes such as histone hypermethylation and transcriptional gene silencing^[18]^.

Genomic and post-genomic research technologies have shifted to focus on biomarker discovery for diagnosis, prognosis and prediction of treatment response to targeted therapies^[6]^. Continuous discovery of cancer subtypes has proven that tumor cell surface markers used in traditional pathology-based classifications and clinical phenotyping cannot capture the full complexity of tumors^[2-4]^. In tumors, different cell types interact with each other in the tumor microenvironment (TME). Immune and stroma cells of TME play an important role in tumorigenesis and development of metastasis^[9,10]^. TME studies revealed that tumors can be classified into inflamed and non-inflamed tumors^[19]^. The first type contains abundant presence of immune cells: T cells, myeloid cells, monocytes, and the second little or no presence of immune cells, especially T cells^[19]^. Fibroblast cell types are part of TEM, and more precisely cancer-associated fibroblasts (CAFs) have pro-tumor functions in breast cancer as they can enhance metastasis^[20]^. The presence of tumor-infiltrating lymphocytes (TILs) in TME is associated with an overall patient good prognosis, better survival and the success of checkpoint immunotherapy^[19]^. Studies performed a multi-omic analysis of Tumor Cancer Genome Atlas (TCGA) datasets have allowed identification at least six immune subtypes across cancer types^[21]^. Finally, stromal cells and immune cells can preserve the properties of cancer stem cells (CSCs), or cancer initiating cells, which are cells that exert multicellular functions in tumor tissue-specific networks and immune resistance^[22,23]^. More important, CSCs display differentiation-state plasticity that allow cancer cells to undergo epithelial to mesenchymal transition (EMT), a process in which cancer cells acquire migratory and invasive properties^[24]^. These results underline the importance of immunophenotyping as a new modality to sub-classify cancers based on their TME^[19,20]^.

The effectiveness of the targeted therapy strongly depends on both the cancer type and molecular features of the individual tumors^[25,26]^. The context-specific impact of molecular features such as somatic alterations and/or copy number events can be measured using diverse high-throughput techniques such as transcriptomics (the number of counts of mRNA molecules) and (phospho) proteomic and transcription factor (TF) activities^[27,28]^. The reverse phase protein array (RPPA) is a high-throughput antibody-based technique, similar to Western blot, to evaluate protein activities in signaling networks^[27,28]^. This functional proteomic analysis can be done in either flash-frozen or formalin-fixed, paraffin-embedded (FFPE) tissue samples. The use of RPPA data for evaluation of functional signatures linking perturbations in down- and up-stream signal transduction pathways might be crucial for personalizing cancer therapies in future^[18]^.

Computational integrative methods that combine genomic and functional cancer phenotypes may better predict those patients who will benefit of the combination therapies^[27,28]^. This system biology approach generally uses statistical/mathematical modeling and supervised machine learning for learning and predict disease similarities from basic and clinical data. Personalized disease subnetworks may be necessary to uncover cancer-related associations, including genotype-phenotype relationships and spatial heterogeneity in the tumor microenvironmental interactions^[4,27,28]^. However, although powerful, the use of these methodologies still requires additional strategies to reveal functionally important biomarkers, which often remains the rate-limiting step in the diagnostic challenge. Here we will discuss these issues using as model the breast cancer tumors.

## Breast cancer subtypes and therapy outcomes

Breast cancer has the highest incidence in women worldwide and is the fifth leading cause of mortality in the globe. Many breast cancer classifications have been proposed according to the invasive characteristics, occurrence, histology and molecular profiling of tumor samples^[29,30]^. Based on their site of occurrence, tumors can be classified as lobular (located at breast lobules) or ductal (at breast ducts). Carcinomas may also arise from invasive epithelial cells (medullary carcinoma), mucus-producing cells (mucinous carcinoma, also called colloid carcinoma), or a subtype of ductal carcinoma *in situ* (DCIS) or invasive ductal carcinoma (tubular carcinoma). The *in situ* to invasive breast carcinoma progression is often caused by interactions among epithelial, myoepithelial, and stromal cells. The progression occurs due to the loss of normal myoepithelial cell function^[31]^.

Cancers derived from luminal cells are the most common types of breast cancer expressing hormone receptors for estrogen receptor (ER), progesterone receptor (PR), or the amplified human epidermal growth factor receptor (EGFR) 2/erythroblastic leukemia viral oncogene homolog 2 receptor (HER2/ErbB2/ERBB2)^[30]^. A high resolution copy-number analyses have confirmed recurrent amplification on chromosomal regions and genes, respectively, found in primary tumors mapping at 8q24 [v-myc myelocytomatosis viral oncogene homolog (avian) (MYC)], 11q13 [cyclin D1 (CCND1)], 17q12 (ERBB2), 20q13 [serine/threonine kinase 15 (STK15)/aurora kinase A], and homozygous deletion at 9p21 [cyclin dependent kinase inhibitor 2A (CDKN2A)]^[32]^. Tumors lacking expression of all three receptors (ER, PR, HER2) are referred to as triple-negative breast cancers (TNBCs), which are tumors most often derived from cells of basal origin. TNBCs display stem cell-like and luminal progenitor-like gene signatures, and frequently have somatic mutations in the TSGs TP53 and PTEN, and a smaller fraction is also mutant for breast cancer 1 (BRCA1). Molecular gene expression profiling has also redefined breast cancer subtypes as luminal A, luminal B, HER2-rich, and basal-like, which roughly parallel the immune-histochemical categories^[33-35]^. The basal and claudin-low subtypes map to the previously designated basal A, and basal B subtypes, respectively^[36]^. In general, the luminal A breast cancer subtype displays mutations in phosphatidylinositol 3-kinase (PIK3CA) (~49%), mitogen-activated protein kinase (MAP3K1) (~14%), GATA binding factor 3 (14%), TP53 (~12%), and MAP2K4 (~12%) and loss of Phosphatase and tensin homolog deleted on chromosome ten (PTEN) (13%), among others. The luminal B breast cancer subtype has mutations in TP53 (~32%), PIK3CA (~32%), MAP3K1 (~5%) and other genes. HER2-overexpressing tumors display representative TP53 mutation and overexpression of other genes, such as growth factor receptor-bound protein 7 (GRB7) and Post-GPI Attachment To Proteins 3. Patients with HER2-overexpressing tumors usually develop brain metastases and additional mutations in fibroblast growth factor receptor 2, PI3KCA and ataxia telangiectasia and rad3-related kinase, homozygous deletion in CDKN2A as well as amplification in Kirsten rat sarcoma viral oncogene homolog (KRAS)^[33-36]^. Breast cancer patients with BRCA1 germline mutation do not express ER, PR, and HER2 and share morphological, clinical, and molecular features and immunohistochemical and cytokeratin expression patterns like basal like breast cancers^[30,37]^.

Immunohistochemistry and/or fluorescence *in situ* hybridization in slides or tissue microarray (TMA) have been used to identify the distinct primary and invasive and non-invasive breast cancer subtypes^[30]^. The subtyping is critical in clinical management of distinct prognoses and predictive responses to endocrine or targeted therapy^[38,39]^. Breast cancers with HER2/ERBB amplification respond to trastuzumab and/or lapatinib, and tumors with mutated or amplified BCR-ABL (breakpoint cluster region C-ABL oncogene 1, non-receptor tyrosine kinase) respond to imatinib mesylate^[40]^. However, although molecular profiling provides important prognostic indicators, breast cancer risk stratification remains a challenge in TNBC cases^[41]^. For instance, the claudin-low subtype was identified as a TNBC subset that is associated with more aggressive tumor behavior and worse prognosis^[41]^.

**Figure 1:**
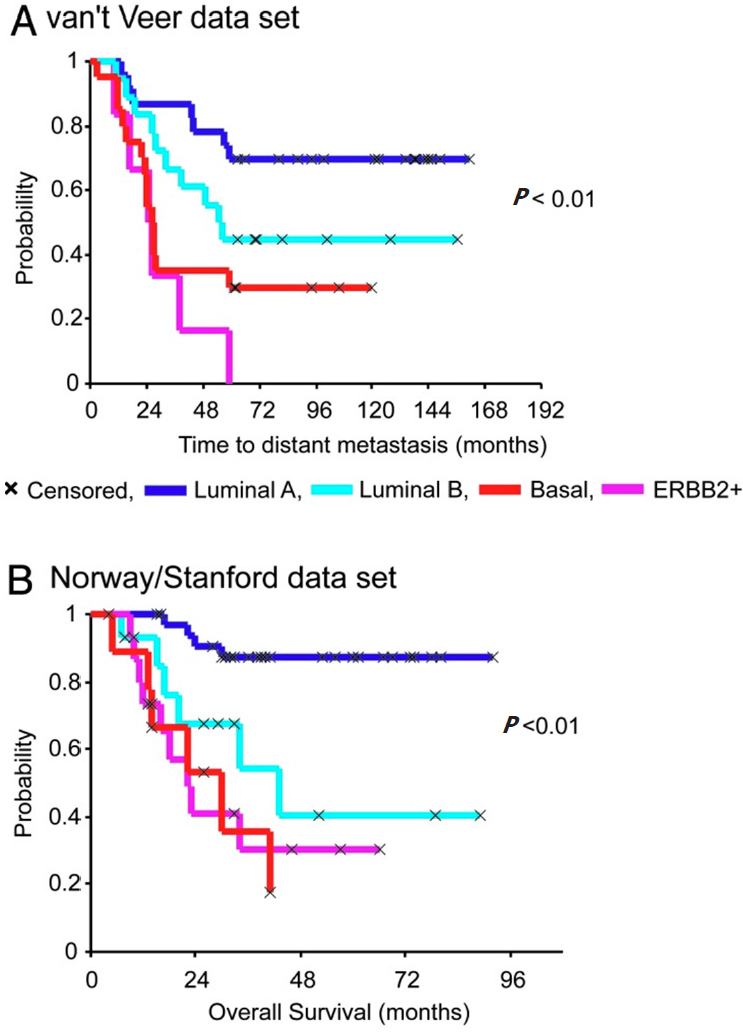
Kaplan-Meier curves of time for distant metastasis (A) and overall survival (B) among five breast cancer subtypes in two patient cohorts. This figure is quoted with permission from Sorlie *et al*.^[33]^

Figure 1 shows the Kaplan-Meier curves that exemplify how hierarchical clustering based on patterns of expression of intrinsic genes or gene expression signatures can discriminate tumors and ultimately the outcomes following the adjuvant and combined systemic treatments^[33]^. Many independent data sets representing different patient cohorts have confirmed the discriminatory power of the molecular subtypes for the representative groups and recommended their treatment as separable diseases^[33]^. Diverse panels of well-characterized breast cancer cell lines have been used to statistically validate the robustness of associations between molecular subtypes and activated signaling pathways to *in vitro* specific therapeutic compound responses^[5-7]^. Surprisingly, more than 50,000 genetic and molecular features emerged from these cell lines which exhibit differential sensitivities to most therapeutic compounds^[5-7]^. Similar mutational profiles have been identified in tumor samples derived from patients’cohorts such as METABRIC (the Breast Cancer International Consortium), TCGA and many other cancer genome cohorts^[42]^. Analyses of subclonal evolution of a cancer cell across lifespan indicated that a dominant common ancestor lineage is present at time of tumor diagnosis^[43]^. Deep analyses of cancer genomes identified over 30 cancer mutational signatures, which are caused by multiple mutational processes include infidelity of either the DNA replication, damage or repair machinery^[43]^. At least six genome rearrangement signatures driving the genomic amplification have been identified in breast cancers^[42]^. These clinical and genomic datasets and somatic mutational catalogues are available online for in silico investigation for their relationships to tumor clinical responses^[42,43]^.

Growing evidence suggests that CAFs is the major cellular component in the peritumoral micro-environment and a strong biomarker for breast cancer growth invasion and dissemination^[20,24]^. CAFs display mesenchymal-like features and are likely mesoderm derived and differ from normal fibroblasts in terms of gene expression profile, activation mechanism and phenotype^[44]^. A study in a Brazilian cohort of breast cancer patients confirmed that CAFs in lymph nodes with macrometastasis express similar profile of vimentin, alpha-smooth muscle actin (-SMA), and S100A4 protein as those CAFs found in primary tumors. CAFs were uniformly ER, PR, HER2, MKI67, and P53 negative, but level of staining of transforming growth factor-1 (TGF-1), CXC chemokine receptor CXCR4, and p-AKT STK(62.3%, 52.4%, 65%, respectively) were equivalent between primary and lymph node metastasis fibroblasts^[44]^. Some anticancer drugs are likely to affect CAFs, however none is known about the impact on therapeutic outcomes.

The presence of distinct global gene-expression signatures within tumors allowed the development of reliable omics-based technologies for clinical diagnostics of breast cancer subtypes^[33-36]^. The Oncotype DX, PAM50, and MammaPrint tests are some examples commercially available^[45]^. The routine application of these tests are useful for identifying stage I or stage II lymph node-negative breast cancer patients that may or may not require adjuvant chemotherapy^[30,46]^. The Oncotype-DX breast cancer assay quantifies the transcription for only 21 genes through reverse transcription polymerase chain reaction using in the breast cancer tissue (lumpectomy, mastectomy, or core biopsy) conserved in FFPE. The Oncotype-DX test provides a recurrence score (from 1 to 100) that is used to predict the risk of recurrence of DCIS breast cancer subtypes and/or the risk of a new invasive cancer^[45]^. The Oncotype DX DCIS test in woman diagnosed with DCIS predicts benefit from radiation after surgery. There is currently clinical practice guideline addressing when biomarker scores might be applied for predicting which women will benefit of specific drugs or regimens for adjuvant systemic therapy^[45]^.

**Figure 2:**
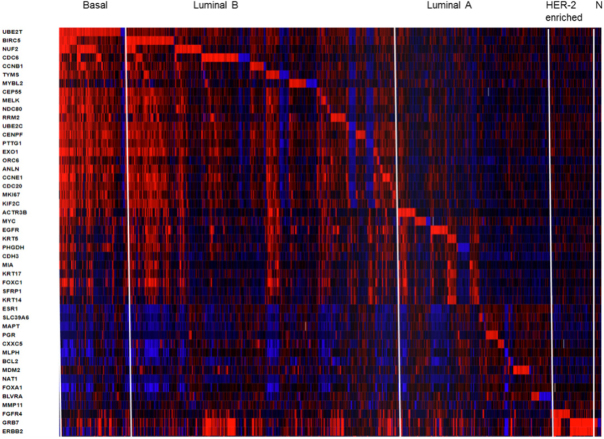
Heatmap of RNA expression (z-scores) of the PAM50 gene set in 1904 breast tumors samples from METABRIC cohort. The visual representation of clustered gene patterns associated with subtypes Basal, Luminal B, luminal A, HER-enriched and Normal-like (left to right order). Each rows correspond to a gene and each column to a patient sample. The data of BAG1, PPR160 and TMEM45B were not included. The heatmap was generated using cBioPortal tools (https://www.cbioportal.org/)

The PAM50 test uses 50 gene expression set to discriminate between each of the 5 breast cancer subtypes. The PAM50 recurrence score estimate the risk of distant recurrence of hormone-receptor-positive breast cancer from 5 to 10 years after diagnosis and after 5 years of hormonal therapy treatment in postmenopausal women^[30,45]^. For illustrative propose we present a heatmap generated in cBioPortal (https://www.cbioportal.org/) for PAM50 gene set in 1904 breast tumors samples from METABRIC study [Figure 2]. This colored representation displays similarities between the tumor samples in terms of messenger RNA expression levels (z-scores) for the panel of genes. The rows correspond to indicated genes and column to samples. The gene clusters that define subtypes Basal, Luminal B, luminal A, HER-enriched and Normal-like markers are separated from left to right order. The PAM50 scores incorporate most highly enriched clusters of genes associated with ER signaling (12 genes), growth factor signaling (4 genes), proliferation (21 genes), invasion (1 gene), basal phenotypes (9 genes) and miscellaneous (3 genes). Cytokeratins KRT5, KRT14 and KRT17, as well as basal markers SFRP1 and MIA differentiates Basal-like from Luminal B tumors. ERBB2, EGFR, FGFR4 and GRB7 differentiates HER2-enriched tumors from other subtypes. The PAM50 and Oncotype DX share the genes MKI67, CCNB1, BIRC5 and MYBL2, which contribute to their proliferation score. The prognostic value of PAM50 is greater than ER/HER2 immunohistochemistry classification^[30,45]^. However, studies have shown that PAM50 score did not improved outcomes of patients who underwent anthracycline- and taxane-based therapy^[30,47]^.

Many predictive modeling methodologies for phenotyping breast cancer subtypes have focused on signaling transduction upon the canonical signaling pathways, including PI3K, mammalian target of rapamycin, MAPK, transforming growth factor β, Wingless/Integrated, cell cycle, apoptosis, immune responsiveness, and DNA damage response pathways^[3,27,28]^. Protein phosphorylation, a reversible post-translational modification at serine (S), threonine (T) and tyrosine (Y) residues, involves a system of sequence-specific kinases (writers), phosphatases (erasers) and reader proteins. The advent of reverse RPPA databases has allowed the phophoproteomic profiling of key cancer-related proteins. The RPPA platform of the University of Texas MD Anderson Cancer Center (https://bioinformatics.mdanderson.org/main/MCLP) displays the protein expression in 650 independent cell lines with known genomic, transcriptomic, and drug-screening data. Analyses in this dataset revealed mutation-induced perturbations of over 200 proteins and their phosphorylated forms in various cancer types^[48]^. The representative nodes and modules of cell network perturbations were used to determine specific clusters for each cancer type. For instance, the breast cancer cluster was distinct from other cancer types by high expression of proteins of androgen hormone receptor canonical pathway and HER2 protein^[48]^. RPPA-based pathway-activation profiling can be a powerful tool to predict putative mechanisms underlying sensitivity and resistance to breast tumor subtypes for specific therapy^[48]^. The phospho-HER2 and phospho-EGFR profiles are particularly important in clinical setting for prescription of EGFR pathway-targeted drugs such as Herceptin, lapatinib and pertuzumab^[40]^.

HER2/ERBB2 is a transmembrane glycoprotein belonging to EGF family of receptors that regulate cell growth, proliferation, survival, differentiation, and angiogenesis^[40]^. Activation of the ERBBs receptors by phosphorylation leads to complex signaling pathways^[40]^. GRB7 is one of the 105 protein coding genes located in the same amplicon as HER2 on chromosome 17q12. Over-phosphorylation of GRB7 in ERBB2 amplified tumors is involved in resistance to anti-HER2 and antiestrogen therapy. A comprehensive gene set enrichment analysis revealed perturbed ERBBs signaling in HER2 amplified breast cancer cells overexpressing dermcidin (DCD), a gene localized at chromosome 12q13 locus and potential oncogene of breast epithelial cells^[49]^. Agreeing with this postulated, we found that DCD is co-expressed with GRB7, ERBB2 and FGFR4 in various cohorts of breast tumor samples, including METABRIC cohort (data not shown). More importantly, DCD has been considered as biomarker for cellular resistance of various tumor cells to the EGFR/ErbB1 tyrosine kinase inhibitors erlotinib and lapatinib^[50]^.

Mutations in PIK3CA gene encoding the p110α catalytic subunit of PI3K, class IA, are among the most common alterations in human malignancies and contribute to approximately 25% of breast cancers^[30]^. Such mutation confers a gain of function to p110α and resistance to HER2-based therapy^[51]^. A combination of three proteins comprising the receptors EGFR, ERBB3/HER3, and the cyclin-dependent kinase inhibitor p27 (CDKN1B) was found to be a potential biomarker for dependence on PI3K/AKT *vs*. MAPK/ERK signaling for drug resistance in HER2 breast cancers^[52]^. A computational algorithm called affinity regression was developed for analysis of distinct dysregulated transcriptional regulators downstream of oncogenic somatic alterations^[53]^. The analyses and validation of the method was done across 12 TCGA cancer data^[53]^. The differential activity of TFs associated to mutant PIK3CA in isogenic breast cancer cell lines was revealed by comparing the phospho (p)-AKT and p-S6K levels^[53,54]^. The results point toward the possible application of these predictive models to screening of drugs to personalized therapies.

Epigenetic studies have found that luminal breast cancers share a common epigenetic signature, whereas basal-like breast cancers display highly heterogeneous signatures^[55,56]^. Basal-like breast cancer cells that constitute the majority of TNBCs can reprogram a subset of luminal breast cancer cells to a basal-like state, which drastically alter their phenotype. This phenotype is associated with high production of the cytokine IL-6 and tumor-promoting molecules^[56]^. Overexpression of three TFs Engrailed 1, T box transcription 18, and T-cell factor 4 can induce the repression of some luminal features in luminal breast cancer cells^[56]^. Over 75% of breast tumors express the ERα that leads to genetic and cellular aberrations^[38,39]^. Patients with ERα-dependent tumor growth often respond well to tamoxifen and fulvestrant^[38,39]^. Nonetheless, patients may develop endometrial cancer and increased bone density. Recent studies using ChIP-seq and chromatin accessibility (DNase-seq and ATAC-seq) assays have identified a unique cistrome ERα profile for breast cancers^[57]^. The TF FOXA1 was found as an essential TF for estrogen binding and induction of ERα-mediated gene expression in breast cancer cells^[58,59]^. Therefore, targeting FOXA1 with small molecules may disrupt ERα positive breast tumor growth. The cyclin D and cyclin-dependent kinases 4 and 6 (CDK4/6) have critical roles in breast carcinogenesis^[29,30]^. Experimental chemotherapy with small molecule inhibitors of CDK/6, such as abemaciclib, palbociclib and ribociclib, have yielded clinical benefits to ER positive breast cancer patients^[60]^. Abenamaciclib decreases cell proliferation and enhances tumor cell intracellular levels of endogenous retroviral genes, triggering “viral mimicry”. This in turn stimulates production of type III IFNs and IFN-induced genes in an autocrine fashion^[60,61]^. Accordingly, treatment of cancer cells with abemaciclib markedly decrease DNMT1 mRNA levels and DNA methylation, therefore, functioning in the similar way as epigenetic modifier 5-azacytidine^[62]^. Finally, epigenetic changes at enhancers and promoters of breast cancer cells as well as tumor stroma cells such as CAFs and myoepithelial cells are critical for transformation and metastasis^[20,24]^. CAFs may also contribute to immune suppressive effect of TME that is one specific biological feature of HER2/neu-positive breast cancers^[63]^. Therefore, DNA demethylating agents and cell cycle checkpoint inhibitors may be promise therapies to breast cancers.

Immunophenopying has been proposed as an biological assay to classify tumors and their microenvironments^[21-23]^. A classification into inflamed and non-inflamed tumors, and presence of the immune cells, especially T cells, have been used as an indicator of clinical response to the immunotherapies^[19-21]^. The immune-inflamed phenotype is rich in immune cells responsive to the immune checkpoint monoclonal antibodies towards CTLA-4 and PD-1, as well as its ligands PD-L1 and PD-L2. These mAbs are highly effective and specific, however, the main drawback of immunotherapies is heterogeneity of response rate, estimated from 10%-40%^[64]^. CD8+ T-cell infiltration increases significantly with tumor mutation load^[64]^. The presence of both PD-L1 expression on breast tumor cells and TILs in TME predict longer disease-free survival and better overall survival to TNBC patients^[65]^. One study investigated the role of PD-1 as a prognostic marker for TNBC in an Asian cohort of 269 patients^[66]^. The results suggested a superior prognostic value of the CD274 (PDCD1 ligand 1, PD-L1) and PDCD1 in TNBC tumor immune microenvironment as compared to classical clinicopathological parameters^[66]^. Overall the results of these studies are providing new insights and alternative routes for possible therapeutic interventions to breast cancers.

An increased number of commensal and pathogenic bacteria, including *Fusobacterium nucleatum*, *Bacteroides fragilis*, *Enterococcus faecalis*, *Streptococcus gallolyticus*, *Helicobacter hepaticus*, and *Porphyromonas gingivalis* have been associated with cancer development. On the other hand, *Faecalibacterium prausnitzii*, *Akkermansia muciniphila*, *Bifidobacterium longum*, *Collinsella aerofaciens* and *Citrobacter rodentium* seem to have an anti-inflammatory and anti-tumoral roles in a variety of tumors^[67]^. A comprehensive analysis of microbiota of 668 breast tumors indicated that H. influenza and *Listeria spp* inhabiting the stromal breast cancer tissue significantly influence the expression of genes in the proliferative pathways: G2M checkpoint, E2F TFs, and mitotic spindle assembly^[68]^. Additionally, one study identified that *Lactobacillus fleischmannii* was associated with epithelial mesenchymal transition^[69]^. Oral supplementation with *A. muciniphila* probiotic restore the efficacy of PD-1 blockade against epithelial tumors by increasing the recruitment of CCR9+CXCR3+CD4+ T lymphocytes in an interleukin-12-dependent manner^[69]^. With the approval of the first chemo- immunotherapy combination for metastatic TNBC^[70]^, we expect that future studies correlating traditionally clinicopathological parameters with additive predictive valor of commensal bacteria will find best cancer response signatures to combination chemotherapy and immunotherapy.

Breast cancer canonical risk factors include age at diagnosis, age at menarche, nulliparity, age at first birth, number of children, months of breastfeeding, race, body mass index, menopausal status, absorption, combined oral contraceptives, age at menopause, prior benign breast disease, and family history (BRCA 1 and 2 and PTEN mutations) of breast cancer^[71]^. The hypothesis that estrogens and estrogen metabolites are implicated in female breast cancer etiology and progression has been confirmed for luminal A and luminal B breast cancer subtypes^[71]^. Environmental factors such as endocrine disruptors compounds (xenobiotics, pesticides and pollutants) may influence mammary and gut microbiota^[72,73]^. However, there is no data to support that a putative disease-causing microorganism or if altered microbial pattern of women could have a role in the development and progression of the breast cancer disease^[72,73]^. The molecular pathological epidemiology (MPE) is a new discipline for investigating specific risk estimative for endogenous and exogenous factors controlling the etiologic heterogeneity of carcinogenic process, as well as environmental and inherited factors leading to failures to pharmaceutical treatment or prevention^[74,75]^. MPE incorporates large multi-omic and epigenomic datasets to estimate the impact of genome-wide association studies on disease entity in population-based cohorts^[74,75]^. MPE methodologies can provide novel insights into mechanistic pathways of common diseases and contribution of medications (pharmaco-MPE), immune mediators, and microorganism (microbial-MPE) on their risk expectative^[75]^. In this way, standardized pathological methods for breast cancer molecular subtyping (whole-tissue specimens or TMA), antibodies for immunohistochemistry, and imaging digital analytical repertories will have great value to establish the multidisciplinary framework for MPE investigations on definitive biomarkers to subtype breast cancers and genotype-phenotype relationships.

## Conclusion

Breast cancer is a highly heterogeneous disease with differences in histopathological and biological characteristics, variable prognoses, and response to therapy. All breast tumor subtypes display different types of clonal subpopulations and this intratumoural and metastatic heterogeneity contribute to different drug sensitivities and resistance characteristics. Since the first study by Sorlie and colleagues in 2003, knowledge about genetic, epigenetic and endogenous and exogenous factors associated with the five subtypes of breast cancers (Luminal A, Luminal B, HER2-enriched, Basal-like and Claudin-low) and their associated aberrant signaling pathways extensively increased and become extremely complex. The multi-omics and MPE methods for large comprehensive molecular cataloguing of cancer patient cohorts will help to establish and predictive biomarkers essential for new therapeutic agents and patients’ responses. A systematic stratification of tumors based on therapeutically actionable mutated gene, TME immune cell profile, mammary microbial profile and epigenetic functional signatures may better predict those patients that will benefit from (neo) adjvuvant multi-agent chemotherapy, targeted and combination therapies, including immunotherapies.

## Declarations

### Copyright

© The Author(s) 2019.
